# Reflections on Health Workforce Development: Comment on "Health Professional Training and Capacity Strengthening Through International Academic Partnerships: The First Five Years of the Human Resources for Health Program in Rwanda"

**DOI:** 10.15171/ijhpm.2018.129

**Published:** 2018-12-25

**Authors:** Gilles Dussault

**Affiliations:** Global Health and Tropical Medicine, Instituto de Higiene e Medicina Tropical, Universidade Nova de Lisboa, Lisbon, Portugal.

**Keywords:** Capacity Development, Health Workforce, Academic Partnerships

## Abstract

This commentary addresses the statement that "the authors believe that the HRH [Human Resources for Health] Program can serve as a model for other initiatives that seek to address the shortage of qualified health professionals in low-income countries and strengthen the long-term capacity of local academic institutions." I adopt the position of the devil’s advocate and ask whether a country, with a profile comparable to Rwanda’s, should adopt this twinning model. I suggest that the alignment with population and other capacity development needs should be the main criteria of decision.

The paper by Cancedda et al^1^ offers an opportunity to reflect on strategies for health workforce development in the context of the pursuit of the health Sustainable Development Goal (SDG-3) and universal health coverage. The authors of this “organizational case study” report on the conception, implementation and results of a twinning initiative designed to increase the production of qualified health workers and to strengthen the capacity of education of health workers institutions in Rwanda. This intervention mobilized considerable financial ($US158 million) and academic (more than 20 US universities) resources in support to 22 training programs in medicine (13 specialized masters), nursing, midwifery, oral health and in “health management and implementation.” The paper describes the process of the twinning and its main results in terms of number of graduates between 2012 and 2017 (n = 3306 and 1300 in the pipeline) and of new faculty trained (24 in medicine and 21 in nursing). Initially, one the objectives was set as to contribute to improving health outcomes through increasing the availability of health workers, but this proved difficult to measure, if only because the time lag is much too short between the arrival of additional health workers on the labour market and changes in health status. Also, as time passes, attribution of whatever observed changes to a specific factor is increasingly difficult given the numerous potential determinants at work. The article also shows the strengths and weaknesses of this capacity strengthening intervention and suggests ways of making it more effective.

This commentary addresses the concluding statement that “the authors believe that the HRH [Human Resources for Health] Program can serve as a model for other initiatives that seek to address the shortage of qualified health professionals in low-income countries and strengthen the long-term capacity of local academic institutions.” I adopt the position of the devil’s advocate and ask whether a country, with a profile comparable to Rwanda’s, should adopt this twinning model.

The first question that comes to mind is whether the model is aligned with the needs of the population. If like in Rwanda the main mortality causes are lower respiratory infections, neonatal disorders, diarrheal diseases and malnutrition among children, and tuberculosis and HIV-AIDS among adults (http://www.healthdata.org/rwanda), a focus on specialty medicine and nursing is a surprising priority. In Rwanda, family medicine and community health, which are the better adapted strategies to address these problems, were “de-prioritized” on the grounds that “new medical graduates opting to join the health workforce as general practitioners (rather than pursue specialty training) were felt to already possess the proper knowledge and skills required to deliver basic medical, surgical, and obstetrical services.” Forty years after the Alma-Ata Declaration,^2^ there is a renewed call to give primary care services a higher priority^3^ and to equip health workers, physicians in particular, with competencies in health promotion, prevention of evitable illness, and treatment of problems such as those mentioned above. Should a poor country with an important deficit of qualified health workers invest its limited resources in training specialists who will likely work in hospitals and in urban areas, when the greatest proportion of health problems require community-based primary care services^ [1] ^? These can be provided by non-physician clinicians, like the clinical officers which Rwanda has been training since 2011 and nurses and midwives, complemented by community health workers.^4^ As to developing the capacity of education institutions to deliver specialty programs, an alternative for a small poor country can be to partner with neighboring countries and divide the task of training specialists.

Another relevant question is to what extent a capacity development intervention should go beyond providing basic training and strengthening education institutions, whose capacity to provide in-service training the initiative did not seem to have been targeted. As much as it is important to increase the availability of health workers by producing more, other aspects of building a performing health workforce are just as important, such as a more balanced distribution of workers by levels of care and by geographical zones, a decent and motivating work environment. Also producing more cannot mean producing more of the same as population needs and corresponding competencies required change. Capacity is needed at institutional level where policy development and regulation take place. For instance, this may mean strengthening data bases and research capacity to inform the decision-making process or developing solid regulatory mechanisms to ensure the quality of education and of practice through accreditation agencies and strong professional councils. Capacity is also needed at the level of provider organizations, not only in terms of better trained managers, but also in terms of structure and processes of management.

A third question is whether a country should diversify sources of support and combine North-South and South-South partnerships to develop its health workforce. Partners, even the best intentioned ones, come with their experience, their traditions, their culture and values, their language and also their interests. In principle, diversification would enable a country to be exposed to a range of visions and of policy options and experiences. When sources of funding are diverse, the probability is higher of capacity development being more sustainable, as vulnerability to a funder’s change of policies is diminished.

In a world without constraints, health workforce development can be guided first by an analysis of the current labour market situation and by an identification of deficiencies that need to be addressed. Then strategies and actions with the best cost-benefit and feasibility prospects can be identified and whoever has the competencies to implement them can be selected to do so. In such a rational world, the process is monitored and corrections are made as necessary. Capacity development is then directed at gaps at institutional, organizational and individual level. In the real world of countries depending on foreign assistance, decisions are constrained by various factors. Multilateral and bilateral funders need to account for how they spend public money and to show results; training programs are often seen as the most visible form of capacity development as it produces short-term measurable effects, in terms of number of activities and persons trained. Vertical programs also find it easier to train their country collaborators to perform specific tasks, rather than wait for national institutions to be able to produce the type of health workers they need.^5^ Governments tend to accept this type of intervention more easily than policy or regulation ones and in the end, both funder and recipient are satisfied. Population needs and impact on health outcomes are not always the main criterion in the design of the capacity development project. Funders have their policies (and interests) and so have governments, and these shape capacity development actions.

The Rwanda case study presents a project that globally was successful. The combined education experience of highly respected US institution was certainly determinant in achieving the objectives of augmenting the availability of certain cadres and of strengthening the capacity of partner institutions. The question here is what kind of support to health workforce development fits the needs of low-income countries with high child and maternal mortality and critical needs-based shortages of health workers.

## Ethical issues

Not applicable.

## Competing interests

Author declares that he has no competing interests.

## Author’s contribution

GD is the single author of the paper.

## Endnotes


[1] The paper mentions the “target” of 2.3/1000 qualified health workers “recommended” by the World Health Organization (WHO) in its *World Health Report 2006: Working Together for Health*. This is a common misinterpretation of the report: the 2.3/1000 doctors, nurses and midwives is the observed threshold below which a country is unlikely to be able to cover its basic maternal and child health needs. In fact, WHO has never recommended health worker/population ratios.

